# Depression in Opioid Use Disorder: A Tripartite Psychopathological Model for Dual Disorders

**DOI:** 10.3390/jcm15145476

**Published:** 2026-07-13

**Authors:** Angelo Giovanni Icro Maremmani, Filippo Della Rocca, Silvia Bacciardi, Silvia Cimino, Luca Cerniglia, Alessandro Pallucchini, Manuel Glauco Carbone, Mario Miccoli, Icro Maremmani

**Affiliations:** 1 Faculty of Medicine, Saint Camillus International University of Health and Medical Sciences (UniCamillus), Via di Sant’Alessandro 8, 00131 Rome, Italy; icromaremmani@gmail.com; 2 VP Dole Research Group, PISA-School of Addiction Medicine, G. De Lisio Institute of Behavioural Sciences, Via di Pratale 3, 56121 Pisa, Italy; filippo.dellarocca@yahoo.it (F.D.R.); silvia.bacciardi@uslnordovest.toscana.it (S.B.); alessandro.pallucchini@uslnordovest.toscana.it (A.P.); manuelglaucocarbone@gmail.com (M.G.C.); 3 Addiction Unit, Department of Mental Health and Addictions, ASL5 Liguria NHS, Via Dalmazia 1, 19124 La Spezia, Italy; 4 Department of Psychiatry and Addictions, Section of Psychiatry, North-Western Tuscany Local Health Unit, Tuscany NHS, Versilia Zone, Via Aurelia 335, 55041 Lido di Camaiore, Italy; 5 Department of Dynamic, Clinical and Health Psychology, Sapienza University of Rome, 00185 Roma, Italy; silvia.cimino@uniroma1.it; 6 Faculty of Psychology, International Telematic University Uninettuno, 00186 Rome, Italy; luca.cerniglia@uninettunouniversity.net; 7 Department of Psychiatry and Addictions, Section of Addictions, North-Western Tuscany Local Health Unit, Tuscany NHS, Apuan Zone, Via Carriona 245, 54033 Carrara, Italy; 8 Division of Psychiatry, Department of Medicine and Surgery, University of Insubria, Viale Luigi Borri 57, 21100 Como, Italy; 9 Department of Human and Social Sciences, Mercatorum University, Piazza Mattei 10, 00186 Roma, Italy; mario.miccoli2020@virgilio.it

**Keywords:** opioid use disorder, dual disorders, bipolar spectrum, substance-induced depression, reward dysfunction, addiction psychopathology, worthlessness/being trapped syndrome

## Abstract

Depressive symptoms are highly prevalent among individuals with substance use disorders, particularly opioid use disorder (OUD), yet their clinical meaning is often difficult to determine. In routine practice, these symptoms are commonly interpreted within a binary framework, either as manifestations of an independent mood disorder or as substance-induced states related to intoxication, withdrawal, or early abstinence. Although clinically useful, this distinction may not fully capture a third configuration in which depressive psychopathology emerges within the chronic addictive process itself. This Perspective proposes a tripartite model of depressive states in OUD informed primarily by clinical and dimensional studies conducted in populations with heroin use disorder. First, substance-induced depression refers to mood symptoms temporally linked to the acute or subacute pharmacological effects of substances and generally expected to improve with stabilization or sustained abstinence. Second, an independent mood disorder may coexist with OUD, with particular attention warranted for bipolar-spectrum presentations characterized by affective instability, cyclothymic or irritable temperaments, mixed features, and mood-related patterns of substance use. Third, addiction-related depression describes a persistent depressive configuration associated with reward dysregulation, hypophoria, motivational depletion, cognitive inefficiency, and the Worthlessness/Being Trapped dimension identified primarily in dimensional studies of heroin use disorder. These configurations should not be regarded as rigid or mutually exclusive categories, but as longitudinal clinical formulations that may predominate at different stages of the addictive trajectory. Distinguishing among them may improve diagnostic clarity and support a hierarchical clinical approach in which stabilization of OUD is generally an initial therapeutic priority, while antidepressant and mood-stabilizing interventions are guided by symptom severity, temporal course, psychopathological organization, and evidence of an independent mood disorder.

## 1. Introduction

Depressive symptoms are among the most common psychopathological manifestations in individuals with substance use disorders (SUDs) and are particularly frequent in opioid use disorder (OUD) [1]. At treatment entry, patients often report dysphoria, anhedonia, reduced motivation, cognitive inefficiency, sleep disturbances, and hopelessness. This symptom burden is associated with poorer psychosocial functioning, increased relapse vulnerability, and greater clinical complexity [2,3,4]. Despite their high prevalence, depressive states in patients with SUDs remain difficult to interpret clinically [5]. The prevailing diagnostic approach generally distinguishes between two broad configurations: an independent mood disorder, leading to a diagnosis of dual diagnosis, and a substance-induced depressive condition related to intoxication, withdrawal, or other pharmacological effects of psychoactive substances. Although clinically useful, this dichotomy does not fully capture the heterogeneity of depressive phenomena observed throughout the course of addiction. Depressive symptoms may arise from a pre-existing affective vulnerability, from acute or subacute substance-related effects, or from the progressive neurobiological and psychopathological consequences of chronic addiction. These mechanisms may overlap within the same patient, yet they differ in their temporal course, clinical meaning, and therapeutic implications. This Perspective proposes a tripartite model for interpreting depressive states in OUD. The first configuration is substance-induced depression, in which mood symptoms are temporally related to intoxication, withdrawal, or early abstinence. The second is an independent mood disorder (bipolar dual depression), in which depressive symptoms occur within an autonomous mood disorder—most notably a bipolar-spectrum condition—coexisting with OUD and therefore fulfilling the conventional definition of dual diagnosis. The third is addiction-related depression, in which depressive psychopathology develops as part of the chronic addictive process itself and is associated with reward dysregulation, hypophoria, motivational depletion, and the Worthlessness/Being Trapped (W/BT) dimension described predominantly in studies of heroin-dependent populations [6,7,8].

The aim is not to introduce a new formal diagnostic category, but to provide a clinically useful psychopathological framework. This model may help clinicians avoid two opposite errors: overdiagnosing an independent depressive disorder when symptoms are primarily related to substance exposure or withdrawal and overlooking an underlying affective vulnerability when depressive symptoms occur within a bipolar-spectrum dual disorder. It may also facilitate the recognition of patients whose depressive state is more appropriately understood as an expression of addiction-related psychopathology rather than as a separate mood disorder.

## 2. Conceptual Approach

This article adopts a narrative and conceptual Perspective approach and is not intended as a systematic review or meta-analysis. It integrates clinical and psychopathological findings from studies on dual diagnosis, substance-induced psychopathology, heroin use disorder, and addiction-related symptom dimensions with broader neurobiological models of reward dysregulation and addiction. Particular attention is given to empirical studies investigating the dimensional structure of psychopathology in heroin use disorder using the Symptom Checklist-90 (SCL-90), as well as to clinical research examining the relationships among OUD, bipolar-spectrum conditions, depressive symptomatology, and opioid agonist treatment. Findings from the broader literature on addiction neurobiology, reward deficiency, stress-system activation, negative reinforcement, and dual diagnosis are used to contextualize this evidence [9,10,11,12]. The purpose of this integrative approach is to develop a coherent clinical model capable of distinguishing depressive states that may appear phenomenologically similar but differ in temporal course, psychopathological organization, and therapeutic priority. Accordingly, the following sections examine three depressive configurations: substance-induced depression, independent mood disorder (bipolar dual depression), and addiction-related depression.

## 3. The Tripartite Model of Depressive States in Opioid Use Disorder

### 3.1. Substance-Induced Depression

Substance-induced depression is the most readily recognizable depressive configuration in patients with SUD. In this condition, mood symptoms are temporally related to intoxication, withdrawal, or early abstinence and closely follow changes in substance exposure. Current diagnostic systems define substance-induced depressive disorders primarily on the basis of this temporal relationship between depressive symptoms and the pharmacological effects of a psychoactive substance [13].

Clinically, substance-induced depression may present with dysphoria, irritability, anhedonia, anxiety, sleep disturbance, and reduced motivation. These symptoms may occur across different substance classes, although their phenomenology, severity, and duration vary according to the pharmacodynamic properties of the substance involved. Stimulant use may be followed by a crash phase characterized by depressed mood, fatigue, and reduced reward responsiveness; alcohol and sedative withdrawal may produce affective instability through alterations in stress systems and inhibitory neurotransmission; and opioid withdrawal is commonly associated with dysphoria, irritability, anhedonia, anxiety, and autonomic discomfort [9,14]. The central diagnostic clue is the temporal course. Symptoms typically emerge during intoxication, withdrawal, or shortly after a period of heavy use and tend to improve as the acute pharmacological effects resolve or clinical stabilization is achieved. Longitudinal observations and meta-analytic evidence indicate that a substantial proportion of depressive symptoms in substance-using populations remit after detoxification or during the early phases of treatment, suggesting that they may reflect transient pharmacological or early recovery-related phenomena rather than an independent mood disorder [4]. This distinction has important clinical implications. Prematurely interpreting substance-induced depression as an independent mood disorder may lead to unnecessary pharmacological treatment, obscure the diagnostic picture, and delay adequate stabilization of the addictive disorder. In OUD, the initiation or optimization of opioid agonist treatment (OAT) may reduce withdrawal-related dysphoria, anhedonia, anxiety, and motivational impairment during the early stages of treatment [15,16]. Antidepressant treatment should therefore generally be considered after reassessment during stabilization, particularly when depressive symptoms persist beyond the expected course of intoxication, withdrawal, or early recovery, while remaining immediately indicated whenever symptom severity, suicidality, or an established independent mood disorder requires specific intervention.

### 3.2. Independent Mood Disorders and Bipolar-Spectrum Dual Depression

Independent mood disorder (bipolar dual depression) refers to depressive states occurring in patients with dual diagnosis, in whom the depressive presentation is embedded within a bipolar-spectrum organization rather than representing an uncomplicated unipolar depressive disorder. In this configuration, depressive symptoms are not strictly linked to intoxication or withdrawal and may persist independently of the acute pharmacological effects of substances. They occur within a broader pattern that may include affective instability, temperamental dysregulation, recurrent mood shifts, impulsivity, mixed features, and mood-dependent patterns of substance use [5]. The distinction between dual diagnosis and substance-induced psychopathology is central. Traditionally, dual diagnosis refers to the coexistence of two independent mental disorders in the same individual. In clinical practice, however, differentiating an independent psychiatric disorder from substance-related manifestations often requires longitudinal observation, because psychoactive substances may produce transient symptoms that mimic primary psychiatric conditions. Diagnostic confidence increases when the psychiatric disorder persists independently of substance exposure or follows a course that cannot be adequately explained by intoxication, withdrawal, or early recovery [17,18]. Several etiological models have been proposed to explain the relationship between psychiatric disorders and SUD. These include the primacy of the psychiatric disorder, the primacy of addiction, shared vulnerability factors, and the possibility that some apparent comorbidities are overestimated because substance-related manifestations are interpreted as independent disorders [19,20,21]. These models are clinically relevant because they discourage a simplistic interpretation of depression in addiction. In a patient with OUD, a depressive state may reflect an independent affective disorder, a substance-induced condition, or a psychopathological manifestation arising from the addictive process itself. Evidence from heroin-dependent populations suggests that anxiety–depressive symptoms at treatment entry are not necessarily predictive of dual diagnosis. In a study of more than 1000 heroin-dependent patients, psychomotor excitement and psychotic symptoms were more strongly associated with dual diagnosis than anxiety–depressive symptomatology [22]. Studies examining the chronological relationship between psychiatric disorders and heroin dependence have also found that chronic psychotic and anxiety disorders frequently preceded OUD, whereas recurrent depressive and bipolar disorders were more often identified after the onset of opioid dependence [19]. These findings complicate a straightforward application of the self-medication hypothesis to affective disorders in OUD. Within this framework, depressive presentations in patients with dual diagnosis should not automatically be interpreted as uncomplicated unipolar depression. In a clinically relevant subgroup, the depressive pole appears to be part of a bipolar-spectrum diathesis. This interpretation is supported by studies linking suicidality in heroin addiction to bipolar-spectrum diagnoses and hostile–depressive symptomatology [23], as well as by observations associating cocaine use in heroin-dependent patients with cyclothymic and hyperthymic temperamental traits [24]. More broadly, the coexistence of bipolar disorder and SUD is associated with greater illness severity, poorer outcomes, and increased clinical complexity [25,26,27]. Temperament studies further support this perspective. Heroin-dependent patients, even in the absence of a formal psychiatric comorbidity, show higher levels of cyclothymic and irritable temperamental traits than controls [28]. This finding suggests that affective instability, particularly in cyclothymic and irritable forms, may represent a shared vulnerability substrate for addiction and bipolar-spectrum conditions [29]. Patterns of polysubstance use may also vary according to mood state. In heroin-dependent patients with bipolar disorder, depressive phases have been associated more frequently with sedative or anxiolytic substances, hypomanic phases with stimulants such as cocaine and amphetamines, manic phases with stimulants and cannabinoids, and mixed states with combinations of sedatives and stimulants [30,31,32]. These observations argue against a unitary interpretation of depression in patients with dual diagnosis and support the clinical relevance of mood-instability frameworks. The therapeutic implications are substantial. When depressive symptoms occur within a bipolar-spectrum dual diagnosis, mood stabilization should take priority over antidepressant activation. Antidepressants may be useful in selected cases, but they should be introduced cautiously and generally after adequate mood stabilization because of the risk of affective destabilization, mixed states, or relapse in vulnerable patients [33,34]. In OUD, this strategy should be integrated with adequate OAT, because stabilization of the addictive disorder may itself improve mood regulation and reduce dysphoric states [35,36]. Although this section emphasizes bipolar-spectrum depression, the tripartite model does not exclude the coexistence of OUD and an independent unipolar major depressive disorder. Whenever a mood disorder fulfills established diagnostic criteria and remains longitudinally independent of the addictive process, it should be conceptualized within dual diagnosis. The emphasis on bipolar-spectrum depression reflects its particular psychopathological relevance in OUD, where affective instability and bipolar-spectrum features may provide a more informative framework for interpreting persistent depressive symptoms than an apparently unipolar presentation [24,37]. Nevertheless, depressive episodes that initially appear unipolar require careful longitudinal reassessment, because they may ultimately prove to be substance-induced or addiction-related rather than expressions of an autonomous mood disorder.

### 3.3. Addiction-Related Depression and the W/BT Dimension

A third depressive configuration is not fully captured by either substance-induced depression or an independent mood disorder. Addiction-related depression refers to a depressive state that emerges as part of the chronic addictive process itself. It reflects the progressive neurobiological and psychopathological consequences of addiction, particularly reward-system dysregulation, stress-system recruitment, hypophoria, and motivational depletion. Addiction-related depression is not proposed here as a validated nosological entity or as a diagnostic category equivalent to major depressive disorder or substance-induced depressive disorder. Rather, it is conceived as a heuristic and dimensional psychopathological construct derived primarily from studies of symptom organization in heroin-dependent populations. Its purpose is to describe a clinically recognizable depressive configuration within the addictive process and to support longitudinal clinical formulation without replacing existing diagnostic categories. Contemporary models describe addiction as a chronic relapsing disorder involving dysregulation of motivational, emotional, and executive-control systems [10,11,38,39]. The transition from impulsive to compulsive substance use is associated with changes in reward learning, salience attribution, inhibitory control, and stress responsiveness [40]. Repeated cycles of intoxication and withdrawal progressively shift substance use from positive reinforcement, driven by pleasure and euphoria, toward negative reinforcement, driven by relief from dysphoria and distress [9,11,41,42]. This transition is accompanied by a progressive alteration in hedonic functioning. In the early stages of addiction, substance use may enhance reward responsiveness and produce transient euphoria. As addiction progresses, these effects are increasingly replaced by reduced reward sensitivity, chronic dysphoria, anhedonia, and motivational depletion. Substance use may then persist not primarily to obtain pleasure, but to alleviate a persistent negative emotional state. Several related constructs have been used to describe this condition. Reward deficiency refers to reduced responsiveness to rewarding stimuli. Hypophoria describes a persistent reduction in the capacity to experience pleasure and emotional well-being. Secondary withdrawal refers to a prolonged post-withdrawal state characterized by reduced motivation, dysphoria, and impaired responsiveness to natural rewards. Hyperkatifeia describes heightened negative emotionality associated with chronic addiction. Together, these processes contribute to the maintenance of substance use through negative reinforcement [11,42,43,44]. Dimensional studies of heroin use disorder have attempted to characterize addiction-related psychopathology beyond conventional psychiatric categories. Factor-analytic investigations using the Symptom Checklist-90 (SCL-90) in large samples of heroin-dependent patients identified five relatively stable dimensions: Worthlessness/Being Trapped (W/BT), Somatic Symptoms (SS), Sensitivity/Psychoticism (S/P), Panic Anxiety (PA), and Violence/Suicide (V/S) [6,45]. These dimensions do not simply reproduce the conventional structure of general psychiatric psychopathology and may represent specific patterns of symptom organization within addictive disorders. The W/BT dimension is particularly relevant to depressive psychopathology. It includes feelings of worthlessness, loneliness, hopelessness about the future, reduced interest, impaired concentration, cognitive inefficiency, and a sense of being unable to complete even simple tasks. Suicidal ideation is not a defining feature of this dimension and is captured separately within the V/S domain [46]. This finding should not be interpreted as suggesting that suicidality distinguishes addiction-related depression from major depressive disorder. Rather, it reflects the specific organization of symptoms within the five-dimensional model, in which suicidal ideation consistently clustered within the Violence/Suicide dimension rather than within the W/BT profile. Comparative studies of patients with heroin use disorder and patients with major depressive disorder further support this distinction. Although patients with major depression may show greater overall psychopathological severity, heroin-dependent patients appear to display a different qualitative organization of symptoms, with W/BT, SS, and S/P being more characteristic of heroin addiction and V/S more strongly associated with primary depressive disorders [47]. Further analyses of W/BT in heroin use disorder describe a configuration characterized by dysphoria, interpersonal sensitivity, cognitive inefficiency, emotional vulnerability, intrusive thoughts, and a persistent sense of being blocked, whereas classical depressive features such as loss of sexual interest appear less prominent [46]. Addiction-related depression shares several features with major depressive disorder, including dysphoria, hopelessness, anhedonia, and reduced motivation. The proposed distinction, however, does not depend on the presence or absence of individual symptoms. It concerns the overall psychopathological organization of the depressive state. In addiction-related depression, cognitive inefficiency, interpersonal sensitivity, a subjective sense of being trapped or blocked, motivational depletion, and reward-system dysregulation become the predominant clinical features. The W/BT profile should therefore be interpreted as a characteristic depressive configuration emerging within the addictive process rather than as an alternative diagnostic category. The main phenomenological similarities and differences between addiction-related depression and major depressive disorder are summarized in Table 1. These findings suggest that some depressive presentations in OUD may be more appropriately understood as addiction-related psychopathology rather than being automatically classified as either an independent mood disorder or a transient substance-induced condition. Although addiction-related depression should not be regarded as a formal diagnostic category, the available evidence supports the identification of a set of clinical and psychopathological anchors that may facilitate its longitudinal recognition and differentiation from other depressive presentations in OUD. These anchors are not intended to establish diagnostic criteria. Their purpose is to support clinical formulation, differential interpretation, and treatment sequencing. The proposed framework is summarized in Table 2. Its main clinical value lies in identifying patients whose depressive symptoms may respond primarily to stabilization of the addictive disorder rather than to immediate antidepressant treatment.

## 4. Clinical Synthesis of the Tripartite Model

**Figure 1:**
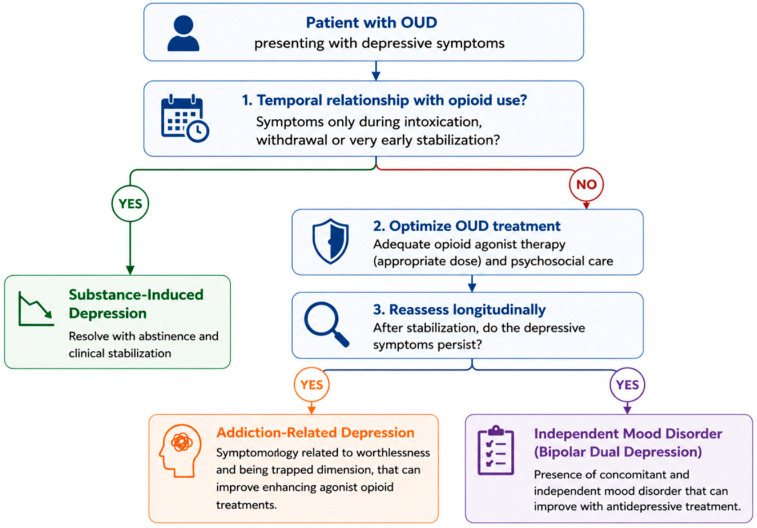
Hierarchical clinical formulation of depressive symptoms in opioid use disorder.

The three depressive configurations can be distinguished according to their clinical context, temporal relationship with substance use, predominant psychopathological profile, and therapeutic priority (Table 3). These configurations should not be understood as three separate depressive disorders necessarily coexisting within the same patient or clinical phase. Rather, they represent different formulations that may become more or less plausible at different stages of the addictive trajectory. The proposed model is therefore hierarchical and longitudinal. In patients with OUD presenting with depressive symptoms, the first clinical task is to assess the temporal relationship between mood symptoms and substance use and to observe their evolution during treatment. Symptoms emerging during active use, intoxication, acute withdrawal, or early abstinence are more likely to reflect substance-induced depression. Depressive symptoms that persist after adequate stabilization, particularly when organized around the W/BT profile, may be more consistent with addiction-related depression. An independent mood disorder should be considered when the depressive syndrome fulfills established diagnostic criteria, follows a course that cannot be explained by substance exposure or the addictive process, or is accompanied by clear bipolar-spectrum features. This sequence is intended to guide diagnostic reasoning and treatment planning rather than to classify patients into rigid or mutually exclusive categories. Stabilization of OUD should generally represent the initial therapeutic priority, followed by reassessment of residual depressive symptoms. Antidepressant treatment remains fully indicated whenever an independent mood disorder or a severe depressive episode is identified according to established psychiatric practice. The same clinical sequence is illustrated in Figure 1.

## 5. Discussion

### 5.1. Positioning the Model Within the Psychopathology of Addiction

The tripartite model proposed in this Perspective is intended to refine, rather than replace, current approaches to depressive symptoms in SUD. Existing diagnostic systems appropriately distinguish independent mood disorders from substance-induced conditions. However, clinical experience and dimensional research suggest that this binary distinction may not fully capture the heterogeneity of depressive presentations in OUD, where mood symptoms may also emerge as part of the chronic addictive process itself [48]. The proposed model should therefore be understood as a clinical and psychopathological framework rather than as a new diagnostic classification. Within this framework, addiction-related depression has an exploratory and dimensional status. It describes a pattern of depressive manifestations associated with chronic addiction-related neuroadaptations but does not constitute a validated nosological entity. Contemporary models of addiction recognize the central role of persistent negative affect, reduced reward sensitivity, stress-system dysregulation, and impaired executive control [9,10,11,12,38,39,40,49]. In these models, dysphoria, anxiety, irritability, and anhedonia are understood as consequences of neuroadaptation and as mechanisms that sustain compulsive substance use through negative reinforcement. Although this framework is essential, affective symptoms are often described as relatively nonspecific expressions of reward- and stress-system dysfunction. The dimensional model derived from studies of heroin use disorder adds a more specific psychopathological level of analysis. It suggests that addiction-related symptoms may be organized into relatively stable and distinguishable dimensions rather than forming a single, diffuse state of negative affect [45]. From this perspective, W/BT is not simply a marker of generic dysphoria. It identifies a depressive configuration characterized by worthlessness, entrapment, cognitive inefficiency, reduced initiative, and impaired motivational drive, whereas suicidal ideation is organized within a separate dimension [46,47]. This distinction is clinically relevant because it allows depressive symptoms to be interpreted according to both their psychopathological organization and their longitudinal course. Transient dysphoria during opioid withdrawal does not require the same clinical formulation as an independent mood disorder occurring within dual diagnosis. Similarly, persistent hypophoria, motivational depletion, and W/BT features after stabilization may not be adequately explained by classical major depressive disorder. The same descriptive label—depression—may therefore refer to substantially different clinical configurations. The tripartite model may also reduce two opposite risks: overmedicalizing dysphoria associated with withdrawal or early recovery, and overlooking a genuine independent affective disorder. Within a hierarchical approach, the first clinical step is to stabilize OUD and observe the evolution of mood symptoms. Residual depressive symptoms should then be reassessed in relation to their temporal course, psychopathological structure, bipolar-spectrum features, and degree of independence from the addictive process.

### 5.2. Pharmacological and Therapeutic Implications

The main therapeutic implication of the tripartite model concerns treatment sequencing. Clinicians frequently face the question of whether depressive symptoms should be treated first in an attempt to reduce substance use, or whether the addictive disorder should first be stabilized in order to clarify the nature of the depressive presentation [50]. The model proposed here supports a hierarchical strategy in which stabilization of OUD with adequate opioid agonist treatment is generally the initial priority. In substance-induced depression, the primary intervention is the stabilization of substance use and the resolution of intoxication, withdrawal, and early recovery-related effects. In OUD, adequate OAT may reduce dysphoria, anhedonia, craving, and motivational impairment during the early stages of treatment [4,16,51]. Evidence for the efficacy of antidepressants in opioid-dependent populations remains inconsistent. Meta-analytic data have found no clear advantage over placebo in some populations [52], whereas other reviews suggest that tricyclic antidepressants may reduce depressive severity but are associated with a greater adverse-event burden [53]. Longitudinal studies have also shown that depressive symptoms may improve during methadone maintenance treatment independently of antidepressant use [54,55,56,57]. These findings do not imply that antidepressants have no role in OUD. Rather, they support reassessment after stabilization, particularly when depressive symptoms persist beyond the expected duration of intoxication, withdrawal, or early recovery. Antidepressant treatment remains appropriate when an independent depressive disorder is established, when symptoms are severe, or when the clinical condition requires immediate intervention. When antidepressants are prescribed during methadone treatment, potential pharmacokinetic interactions should also be considered. Fluoxetine, fluvoxamine, and sertraline may increase methadone concentrations, and fluvoxamine has been associated with substantial increases in methadone plasma levels in some patients [58,59,60,61]. In independent mood disorder (bipolar dual depression), OAT stabilization remains essential, but the treatment of the mood disorder should be guided by its bipolar-spectrum organization. A history of hypomania or mania, cyclothymic or irritable temperament, mixed features, affective lability, impulsivity, and mood-related changes in substance use should increase diagnostic suspicion. In these cases, mood stabilization should precede antidepressant treatment. Antidepressants may be considered selectively, but they should generally be introduced only after adequate stabilization because of the risk of affective destabilization, mixed states, or relapse [33,34]. OAT may also contribute to the reduction of dysphoria and behavioural instability, but it does not replace specific treatment of an independent mood disorder [35,36]. In addiction-related depression, the primary therapeutic target is the stabilization of the addictive disorder and the restoration of functional balance within reward and stress systems. OAT with methadone or buprenorphine may improve depressive symptoms by reducing withdrawal, craving, hypophoria, and negative reinforcement [4,16,62]. Research on the endogenous opioid system also suggests that μ-opioid receptor signaling, κ-opioid receptor-mediated dysphoria, and δ-opioid receptor mechanisms may be relevant to depressive states characterized by anhedonia and motivational depletion [63,64,65,66,67,68,69,70]. Studies of methadone, esmethadone, d-methadone, and related compounds in treatment-resistant depression provide additional, although still preliminary, evidence that opioid-system modulation may influence depressive symptomatology [64,66,71,72,73,74]. These observations should not be interpreted as a general recommendation to use opioid agonists to treat depression outside addiction medicine. Rather, they indicate that in patients with OUD, effective treatment of the addictive disorder may itself reduce depressive symptoms by improving reward regulation, reducing hypophoria, and interrupting negative reinforcement. In addiction-related depression, antidepressants may be introduced when clinically indicated, but they should not substitute for adequate OAT or for stabilization of the addictive disorder.

### 5.3. Diagnostic and Research Implications

The proposed model requires further empirical validation. Substance-induced depression and independent mood disorders are supported by extensive clinical literature and by current diagnostic systems. By contrast, addiction-related depression should presently be regarded as a conceptual and clinical and psychopathological framework derived primarily from studies of heroin-dependent populations. The underlying five-dimensional model has shown substantial stability across different treatment settings, stages of illness, and clinical samples over more than a decade of investigation. Nevertheless, external validation remains necessary before addiction-related depression can be considered a reliably identifiable clinical construct. Future studies should determine whether the three depressive configurations can be distinguished consistently through structured clinical interviews, symptom rating scales, dimensional psychopathological instruments, and longitudinal follow-up. Research should focus particularly on the temporal relationship between depressive symptoms and substance use, the persistence of symptoms after adequate OAT stabilization, the presence of bipolar-spectrum features, and the stability and predictive value of the W/BT profile.

## 6. Conclusions

Depressive symptoms in OUD should not be considered a homogeneous clinical entity. The same clinical presentation—including dysphoria, anhedonia, reduced motivation, and hopelessness—may reflect different underlying configurations. It may be substance-induced and expected to improve with stabilization; it may occur within an independent mood disorder, particularly bipolar dual depression; or it may emerge as part of the chronic addictive process itself, in association with reward dysregulation and the W/BT dimension. Recognizing these distinctions has direct therapeutic implications. Evidence derived primarily from heroin-dependent populations suggests that the first clinical priority is to stabilize OUD and then reassess depressive symptoms longitudinally. Mood-stabilizing treatment becomes central when bipolar-spectrum features are present, whereas antidepressants should be considered selectively, particularly when depressive symptoms persist despite adequate stabilization or when an independent depressive disorder is clinically established. The central clinical question is therefore not only whether depression is present in OUD, but which depressive configuration is being observed. The proposed tripartite model may help clinicians address this question more precisely and support a more rational, pathophysiology-informed approach to dual diagnosis. It is intended to complement, rather than replace, existing diagnostic systems by strengthening longitudinal psychopathological formulation and treatment sequencing in patients with OUD.

## Figures and Tables

**Table 1:** Clinical and phenomenological features that may help recognize the W/BT profile in comparison with the classical psychopathology of major depression.

Phenomenological Domain	Predominant Features of the W/BT Profile	Typical Presentation in Major Depression
Core subjective experience	Feeling trapped, emotionally overwhelmed, unable to move forward despite the desire to do so	Persistent depressed mood and pervasive sadness
Motivational profile	Feeling blocked, reduced initiative, difficulty completing even simple tasks	General loss of interest and reduced goal-directed activity
Cognitive experience	Cognitive inefficiency, poor concentration, “mind going blank”, subjective feeling of reduced mental effectiveness	Negative cognitions, pessimism, self-devaluation, hopelessness
Interpersonal experience	Heightened interpersonal sensitivity, feeling easily hurt, loneliness despite relationships	Social withdrawal mainly related to depressed mood and loss of interest
Relationship with reward	Hypophoria and reduced responsiveness to natural rewards, closely linked to addiction-related reward dysregulation	Anhedonia as part of the depressive syndrome
Suicidal ideation	Not a defining feature of the W/BT dimension (captured separately within the Violence/Suicide dimension)	May be present, particularly in severe depressive episodes

**Table 2:** Proposed Clinical and Psychopathological Anchors for Addiction-Related Depression in Opioid Use Disorder.

Domain	Proposed Clinical and Psychopathological Anchors
Context	Chronic or relapsing opioid use disorder.
Temporality	Depressive symptoms persist beyond acute intoxication, acute withdrawal, or the early stabilization phase.
Phenomenology	Predominance of the Worthlessness–Being Trapped (W/BT) dimension, characterized by hypophoria, motivational depletion, reduced initiative, cognitive inefficiency, feelings of worthlessness, interpersonal sensitivity, and a pervasive sense of being trapped.
Reward profile	Reduced responsiveness to natural rewards, persistent dysphoria, and motivational impairment consistent with chronic reward-system dysregulation.
Differential diagnosis	The clinical picture is not better explained by acute substance-induced depression, independent major depressive disorder, bipolar-spectrum depression, or isolated protracted withdrawal.

**Table 3:** Clinical interpretation of depressive symptomatology in OUD.

Clinical Domain	Substance-Induced Depression	Addiction-Related Depression	Independent Mood Disorder (Bipolar Dual Depression)
Clinical context	Active use, intoxication, withdrawal, or early abstinence	Chronic or relapsing addictive process	Independent psychiatric disorder coexisting with OUD
Temporal relationship	Closely linked to substance exposure and expected to improve with stabilization	Persists beyond acute withdrawal and early stabilization	Persists independently of substance use and the addictive course
Predominant profile	Acute affective and withdrawal-related symptoms	W/BT profile, hypophoria, motivational depletion, and reward dysregulation	Established mood syndrome, often with bipolar-spectrum features
Initial clinical priority	Stabilize OUD and resolve acute substance-related effects	Optimize OAT and reassess reward-related and motivational symptoms	Stabilize OUD with OAT first and introduce antidepressant or mood-stabilizing treatment according to the independent mood disorder
Role of longitudinal reassessment	Confirm symptom remission or persistence after stabilization	Assess persistence and psychopathological organization after stabilization	Confirm longitudinal independence and bipolar-spectrum features

## Data Availability

No new data were created or analyzed in this study. Data sharing is not applicable to this article.
